# Comparative Assessment of Synthetic and Algal-Derived Astaxanthin Supplementation on Growth Traits, Lipid Homeostasis, and Liver Well-Being in High-Fat Diet-Fed *Trachinotus ovatus*

**DOI:** 10.1155/anu/2756729

**Published:** 2025-10-29

**Authors:** Yucai Guo, Haiqi Pu, Sihan Lin, Baoyang Chen, Anqi Chen, Mingyan Huai, Wei Zhao, Jin Niu

**Affiliations:** ^1^State Key Laboratory of Biocontrol, Guangdong Provincial Key Laboratory for Aquatic Economic Animals and Southern Marine Science and Engineering Guangdong Laboratory (Zhuhai), School of Life Sciences, Sun Yat-Sen University, Guangzhou 510275, Guangdong Province, China; ^2^BASF (China) Co., Ltd., Pudong, Shanghai 200137, China

**Keywords:** astaxanthin, fat deposition, immune resilience, lipid metabolism, oxidative defense

## Abstract

While aquaculture often relies on excessive lipid intake to bolster fish growth and cut feed expenses, this practice can also result in hepatic fat accumulation, inflammatory responses, oxidative damage, and immune system malfunctions in aquatic creatures. Astaxanthin (AST), a potent antioxidant, holds promise in mitigating these detrimental effects associated with high-lipid (HL) diets. The objective of this study was to investigate the functional contributions and the fundamental molecular pathways of both synthetically produced and algae-derived Ast, focusing on their impacts on growth rates, lipid regulation, and hepatic well-being in juvenile *Trachinotus ovatus* maintained on diets rich in lipids. The experimental setup involved feeding *T. ovatus* for 8 weeks with four different diets: a control feed (CF) with normal lipid content, a HL diet, a HL diet supplemented with synthetic AST (HL + S), and a HL diet supplemented with algal-derived AST (HL + A). When compared to the HL diet, the inclusion of Ast from both sources in the feed significantly improved the growth performance and survival rate (SR) of *T. ovatus*. Furthermore, both forms of Ast significantly increased glutathione reductase (GR)activity and high-density lipoprotein (HDL) levels, while also decreasing the levels of lipid transport-related substances in the serum. They were also successful in mitigating hepatic lipid overload. Regarding antioxidant potential, the addition of Ast notably potentiated the Nrf2/Keap-1 signaling cascades and boosted the functionality of antioxidant enzymes. Furthermore, Ast from both synthetic and algae origins augmented the innate immune defenses of *T. ovatus*, leading to a decreased sensitivity to stress in the fish. To conclude, incorporating Ast from either source into the high-fat diet of *T. ovatus* mitigated the detrimental consequences of such a diet, including impeded growth, weakened antioxidant defenses, and weakened innate immune responses. Moreover, both sources of Ast exhibited beneficial effects on sustaining lipid metabolism homeostasis and enhancing hepatic well-being in the fish.

## 1. Introduction

Lipids are crucial nutrients for fish growth, playing a significant role in both metabolism and immunity [1]. They provide essential fatty acids and serve as a vital energy source for the growth and overall health of aquatic animals [2]. However, with the decline in global wild fish stocks, coupled with environmental pollution and other challenges, fishmeal production has drastically decreased, leading to a sharp rise in its cost. High lipid (HL) diets have emerged as a promising solution, as they can conserve protein, lower production costs, and reduce reliance on fishmeal. Consequently, HL diets have become a focal point of research in the aquafeed industry [3]. HL diets, though beneficial for energy provision, pose significant risks to the metabolic health of fish [4]. The accumulation of excess lipids in the liver can result in hepatic steatosis, which disrupts normal liver function and impairs the overall health of the fish. Furthermore, the oxidative stress generated by lipid peroxidation can damage cellular components, leading to increased apoptosis and inflammation, which in turn compromise immune function and increase susceptibility to diseases [5]. These adverse effects underscore the need for effective dietary interventions that can mitigate the negative impacts of HL diets. Astaxanthin (AST) supplementation has emerged as a promising strategy to address these challenges.

To tackle the challenges posed by high-fat diets, the identification of effective feed additives is paramount. AST, a well-acknowledged xanthophyll carotenoid celebrated for its robust antioxidant capabilities, has attracted significant attention within the realm of aquaculture due to its multifaceted benefits, encompassing anti-inflammatory, antioxidant, antidiabetic, anticancer, antiaging, and immunomodulatory activities. Additionally, this versatile compound has found extensive application in health supplements, the food processing sector, agricultural endeavors, pharmaceutical preparations, and cosmetic formulations [6, 7]. Moreover, studies have shown that AST, whether synthetic or derived from natural sources such as *Haematococcus pluvialis*, can significantly counteract the oxidative and inflammatory stresses induced by HL diets [8]. Synthetic AST has been observed to enhance the expression of anti-inflammatory genes, such as those involved in the Nrf2-antioxidant response element (ARE) signaling pathway, and to reduce the levels of pro-inflammatory cytokines like TNF-α and IL-6 in the liver [4, 9]. This anti-inflammatory effect helps to protect the liver from damage and maintain its metabolic functions under the stress of a HL diet [10].

The golden pompano (*Trachinotus ovatus*) is a prominent marine carnivorous fish in aquaculture. It holds a prestigious position because of its rapid growth rate, high market demand, and excellent adaptability to various salinity and temperature conditions [8]. Although the optimal lipid concentration of *T. ovatus* is relatively high (i.e., 12%–18%), a HL diet can also lead to several metabolic challenges, including excessive lipid deposition, oxidative stress, and chronic inflammation [3]. Moreover, incorporating AST into the diet of *T. ovatus* has been noted to alleviate the detrimental effects of environmental stressors on its lipid metabolism, thereby preventing growth impediments, liver damage, and exhibiting anti-inflammatory properties [11]. However, the discrepancy in precise molecular mechanisms of synthetic and algal-sourced AST by which they regulate lipid metabolism, reduce inflammation, oxidative stress, and enhance immune and antioxidant capacity remains unclear. This study aims to evaluate the effects of a HL diet supplemented with two sources of AST on the growth performance, lipid metabolism, antioxidant capacity, liver health, and body immunity of *T. ovatus*. The findings will help elucidate the different roles of synthetic and algal-sourced AST in lipid metabolism and its protective effects on fish health.

## 2. Materials and Methods

### 2.1. Diet Preparation and Dietary Treatments

For the experimental trial, we formulated four experimental diets to assess the effects of AST from different sources on HL supplementation on *T. ovatus*. The formula and nutritional composition of the four diets, named a control diet (CF, 11% crude lipid level), a HL diet (HL, 17% crude lipid level), a HL diet that was supplemented with 0.02% synthetic AST (HL + S), and a HL diet that was supplemented with 0.04% algal-sourced AST (HL + A) were listed in Table 1. Dietary synthetic AST was purchased from Baden Aniline and Soda Factory (BASF Co., Ltd., Shanghai, China), and algal-sourced astaxanthin was obtained from Beijing Gingko-Group Biological Technology (BGG Co., Ltd., Beijing, China). The content of AST in synthetic AST is 10%, while that in algae-sourced AST is 5%.

Diets were prepared and processed following previously standard procedure [12]. All dry ingredients were finely ground and sieved through a 60-mesh screen. Ingredients were mixed thoroughly using a high-speed mixer. Soybean oil was mixed with fish oil until homogeneous, then combined with the dry mixture. Distilled water was added to the mixture to achieve appropriate consistency, and the mixture was then extruded into 2.5 mm diameter pellets using a twin-screw cooking extruder (HQ, Zhaoqing, China) with an extruding temperature at 121°C. Pellets were immediately dried into 25°C so as to reduce moisture content, then they were stored at −20°C until required.

### 2.2. Animal Rearing and Experimental Procedures

*T. ovatus* originated from a commercial breeding center situated in Zhangzhou, China. Prior to the commencement of the experimental procedures, these fish underwent a 2-week acclimatization period within the experimental setting, utilizing a standardized control diet to ensure their adaptation. Before the feeding trial commenced, the fish underwent a 24 h fasting period to guarantee an empty digestive system. Subsequently, a total of 640 fish, with an average initial weight of 4.19 ± 0.19 g, were randomly distributed into 16 sea cages (each measuring 1.0 × 1.0 × 1.5 m) at a density of 40 fish per cage. Each experimental diet was assigned to four cages in a randomized manner. The fish were fed their respective diets twice daily, at 08:00 and 15:00, until they reached apparent satiation, for a period of 8 weeks. Throughout this period, daily feed intake and mortality rates were meticulously documented to evaluate growth performance indicators, including weight gain, specific growth rate (SGR), and feed conversion ratio. Throughout the experimental period, water temperature ranged from 25 to 27°C, dissolved oxygen was around 6.01 mg/L, salinity was around 28–31 g/L.

### 2.3. Sample Collection

At the conclusion of the 8-week feeding trial, fish were fasted for 12 h before sampling to ensure an empty digestive tract. Initially, all the fish in the cages were picked up and loaded into small buckets in which 100 mg/L of MS-222 (tricaine methanesulfonate) had been dissolved to minimize stress and ensure humane handling, and the loss of motor ability was taken as the criterion for anesthesia. Then, all fish were weighed and counted for finally body weight and survival rate (SR). Following anesthesia and weighing, the fish were dissected on ice. Thereafter, a total of 10 fish were randomly selected, four for whole-body composition analysis and six for whole-body weight, length, biochemical, and molecular analysis. Blood samples were collected from the caudal vein using sterile syringes prerinsed with heparin to prevent coagulation, and approximately 1–2 mL of blood was collected from each fish. Portions of liver tissues were fixed in 4% paraformaldehyde for 24 h in order to make histological sections. Remaining portions of the liver and head–kidney tissues were immediately frozen in RNA later and stored at −80°C. These samples were later used for the extraction of RNA, analysis of enzyme activity, and gene expression related to lipid metabolism, antioxidant defense, immunity, and inflammation. Finally, fourth of the fish liver in each cage was collected for the detection of protein expression related to lipid metabolism.

### 2.4. Chemical Analysis of Feed and Body Composition

The experimental diets' chemical constituents, comprising moisture, crude protein, and crude lipid, were determined through the application of AOAC methodologies [13]. To determine the crude protein content, Dumas' combustion technique was implemented utilizing the N pro (DT Ar/He Basic) analyzer manufactured by Gerhardt GmbH & Co. KG (Germany), with the formula nitrogen content multiplied by 6.25 yielding the crude protein value. Concurrently, the Soxhlet extraction method was utilized to determine crude lipid content using the Soxtec System HT6 (Tecator). Prior to analysis, the samples were oven-dried at 105°C to ascertain their dry weight and moisture content. Tissue total lipid extracts were processed via a modified Folch method for total lipid extraction. Docosatrienoic (22:3*n−*3) methyl ester (Nu-Chek Prep) was incorporated as an internal standard to quantify the concentrations of individual and total fatty acids. Ultimately, fatty acid profiles of the muscle were quantified using a gas chromatograph (6890 N GC, Agilent Technologies, USA), in accordance with the methodology outlined by Lin and Mui [14].

### 2.5. Enzyme Activity and Hematological Biochemical Index Assays

Approximately 0.1 g of midgut or liver samples were meticulously weighed and then homogenized with 0.9 mL of ice-cold normal saline, diluted to a concentration of 10%. This mixture was subsequently centrifuged at 3000 revolutions per minute (rpm) for 10 min at 4°C using a refrigerated centrifuge (Centrifuge iCEN-24R, ALLSHEGN, China) to separate the homogenate and obtain the supernatants. The supernatants from hepatic and intestinal tissues, were used for detecting enzyme activities. To evaluate the antioxidant status, several parameters were measured using commercial assay kits, including the Catalase (CAT) activity kit (A007-1-1), the Total Antioxidant Capacity (T-AOC) kit (A015-3-1), and the Malondialdehyde (MDA) kit (A003-1-2), all purchased from the Nanjing Jiancheng Bioengineering Institute in Nanjing, China. Additionally, superoxide dismutase (SOD) activity was measured using the S0109 assay kit from Beyotime Biotechnology, following the provided protocol.

Serum biochemical parameters were assessed using a colorimetric method and a Synchron CX5 PRO Clinical System (Beckman Coulter, America). The parameters measured included glutathione reductase (GR), total triglycerides (TGs), total cholesterol (TC), low-density lipoprotein cholesterol (LDL-C), and high-density lipoprotein cholesterol (HDL-C). The assays were conducted with the support by Huili Biotech Co., Ltd., based in Changchun, China.

### 2.6. Hepatic Morphology

Following sample collection, the tissues were promptly fixed in 4% paraformaldehyde and subsequently subjected to a series of graded ethanol dehydration steps (70%, 80%, 90%, 95%, and 100%) to remove moisture. This process was followed by clearing with xylene prior to embedding the tissues in paraffin. The paraffin-embedded blocks were then sectioned into thin slices of 5 μm using a precision microtome (Leica RM2016, manufactured by Leica Microsystems in Wetzlar, Germany). Additionally, to visualize and quantify lipid accumulation, frozen hepatic sections were stained with Oil Red O. Furthermore, to assess DNA integrity, we performed a combined staining protocol utilizing TdT-mediated dUTP Nick-End Labeling (TUNEL) and 4′, 6-diamidino-2-phenylindole (DAPI) using a Fluorescein (FITC) TUNEL Cell Apoptosis Detection Kit (G1501-50T; supplied by Servicebio Co., Ltd., Guangzhou, China). The meticulously prepared tissue slices were examined under either a light microscope or a fluorescence microscope system (Eclipse Ni-E, Nikon Corporation, Japan), and photographic images were captured for further experimental analysis.

### 2.7. Quantitative Real-Time PCR Analysis

Total RNA was isolated from liver and head–kidney tissues using Trizol reagent (Invitrogen, Carlsbad, CA, USA), adhering strictly to the manufacturer's instructions. The concentration and purity of the RNA were evaluated using a NanoDrop spectrophotometer (with an optimal A260:A280 ratio of ~1.90–2.10), while its integrity was confirmed via agarose gel electrophoresis. Subsequently, 2 μg of total RNA were utilized to synthesize cDNA through reverse transcription, employing the PrimeScriptTM RT reagent kit (Takara Bio, Kusatsu, Shiga, Japan). Prior to this step, genomic DNA contamination was removed using gDNA Eraser. The reverse transcription reaction was conducted in a PCR machine, following our standard protocol [3].

To assess the expression levels of target genes, real-time PCR was conducted using a high-throughput, plate-based real-time PCR system (Roche Applied Science, Basel, Switzerland) in conjunction with a highly sensitive reaction mixture composed of SYBR Premix Ex Taq II (Takara Bio, Kusatsu, Shiga, Japan). Each reaction mixture comprised 5 μL of SYBR Premix, 0.2 μL of each primer (10 μM concentration), 4 μL of cDNA template, and 0.6 μL of RNase-free water, totaling 10 μL per reaction. The thermal cycling conditions for the PCR reactions were based on the protocol outlined by Zhao [8]. To confirm the amplification's specificity, a melting curve analysis was conducted. Subsequently, the genes' relative expression levels were determined employing the 2^−ΔΔCt^ approach. The primers utilized in this study are detailed in Table 2.

### 2.8. Western Blotting Analysis

Phospho-ACC (*p* ACC) (11818S), acetyl-CoA carboxylase (ACC) (3676S), Adenosine 5′-monophosphate-activated protein kinase (AMPK) (5831S), and phospho-AMPK (*p* AMPK) (2535S) antibodies, CPT1 (Carnitine palmotoyl transferase 1) (12252S), FAS (Fatty acid synthase) (4233S) and β-actin (4970S) were purchased from Cell Signaling Technology (CST), and HADH (Hydroxyacyl Coenzyme A Dehydrogenase) (19828-1-AP) was sourced from Proteintech (Wuhan, China).

The hepatic tissues were placed on ice and treated with RIPA buffer (1:100 w/v) containing 1% phosphatase inhibitor and protease inhibitor. The tissues were then homogenized for 1 min using a high-throughput tissue crusher for five times. After preparing the liver into a homogenate, the samples were centrifuged at 12,000 rpm for 15 min at 4°C using a cryocentrifuge. The precipitation obtained by centrifugation was collected, and the albumen concentration of which was detected by making use of a BCA kit. The protein samples, separated on 10% and 7.5% SDS-PAGE gels, were transferred to polyvinylidene fluoride (PVDF) membranes. The target proteins were incubated with antibodies in a 5% skim milk solution prepared with TBST buffer. After blocking nonspecific binding, the intensity of the interest protein bands was disclosed after dropwise adding an enhanced chemiluminescence developer and exposing under a multifunctional fluorescence gel imaging system.

### 2.9. Statistical Analysis

All data were expressed in the form of mean ± standard error (SEM) and were processed using SPSS software (version 22.0, IBM Corp., Armonk, NY, USA). Graphical representations of data were created using image forming tool (version 10.1.2, GraphPad Software, San Diego, CA, USA) to visualize the results clearly. An ANOVA method was conducted to estimate the significant differences among the four treatment groups. Duncan's multiple range test was followed used for post hoc comparisons to identify which specific groups differed from each other when a significant difference was shown (*p* < 0.05).

## 3. Result

### 3.1. Growth Performance

The growth performance and feed utilization of *T. ovatus* subjected to diets with and without enriched lipid content and AST supplementation are detailed in Table 3. A significant decrement in weight gain ratio (WGR) and SGR was observed in the HL group compared to the control feed (CF) group (*p* < 0.05). Conversely, no statistically significant differences were discernible among the HL supplemented with AST (HL + S, HL + A) and CF groups in terms of WGR and SGR (*p* > 0.05). Notably, the HL + S group exhibited the highest SR among the four experimental groups. Furthermore, a concerted and pronounced elevation in visceral somatic index (VSI) and hepatosomatic index (HSI) was observed in fish fed the HL diet compared to those receiving the CF diet (*p* < 0.05). However, the supplementation of AST to the HL diet mitigated these increases, resulting in a reversal of the trends observed for VSI and HSI.

### 3.2. Fatty Acid Composition of Muscle

Fatty acid composition of muscle was assayed and summarized in Table 4. As is shown in the result, fish fed the HL diets, including HL, HL + S, and HL + A groups, resulted in increased levels of polyunsaturated fatty acids ∑(PUFAs) such as *n−*3 and *n−*6 fatty acid in muscle, especially in HL + A group. What's attract us most was that the rate of *n−*3/*n−*6 in muscle was diminished sharply in fish fed with HL diet, while it soars in HL + A group compared with CF (*p* < 0.05).

### 3.3. Serum Biochemical Parameters

**Figure 1:**
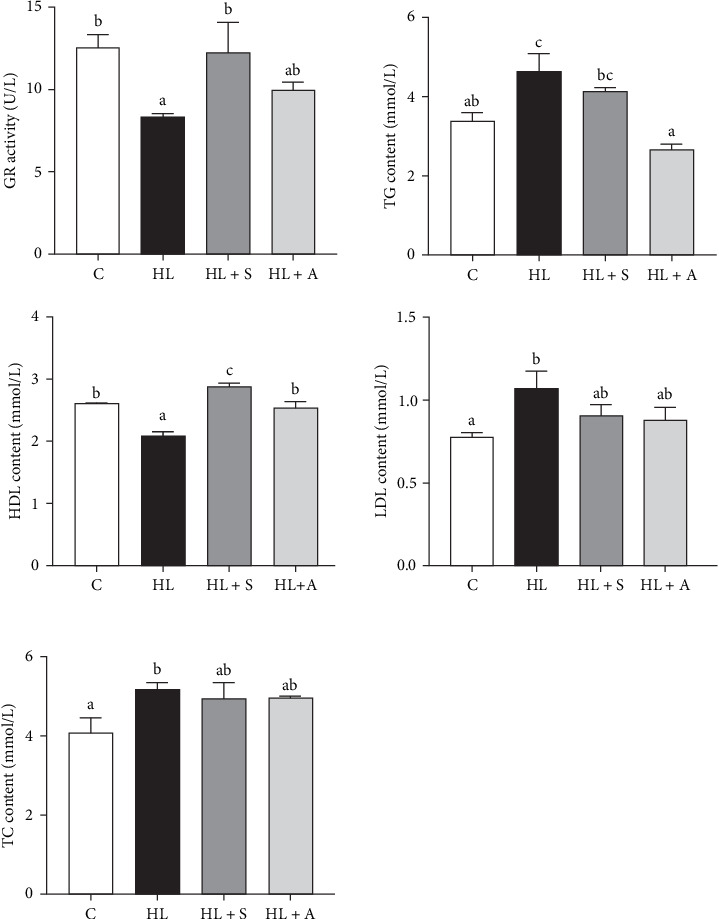
Effects of astaxanthin from different sources on the serum biochemical parameters of *T. ovatus* under a high lipid diet. GR, glutathione reductase; GLU, glucose; HDL, high-density lipoprotein; LDL, low-density lipoprotein; TC, total cholesterol; TG, total triglyceride. Results were presented as “mean ± SEM” of four replicates. Means with different superscripts letters are significantly different (*p* < 0.05).

The serum biochemical parameters are illustrated in Figure 1, revealing notable differences among the experimental groups. Specifically, the levels of GR and high-density lipoprotein (HDL) in serum were significantly reduced in the HL group compared to the CF group (*p* < 0.05). Conversely, no significant variations were observed in GR and HDL levels among the CF, HL + S and HL + A groups (*p* > 0.05). Notably, the HL group exhibited the highest concentrations of TC and TG among the four groups. Additionally, the low-density lipoprotein (LDL) content in fish fed the HL diet surged significantly compared to those fed the CF diet (*p* < 0.05), whereas no discernible differences were detected in LDL levels between the CF, HL + S, and HL + A groups (*p* > 0.05).

### 3.4. Lipid Accumulation and Metabolism Analysis

To delve into the efficacy of AST in mitigating excessive hepatic lipid accumulation, an Oil Red O staining experiment was conducted. The results unequivocally demonstrated that both synthetic and algal AST were capable of effectively reducing both the number and size of lipid droplets in liver tissue, which had initially arisen due to a HL diet. This notable reduction in lipid accumulation points to a protective effect against hepatic steatosis, potentially mediated by AST's ability to modulate lipid metabolism.

**Figure 2:**
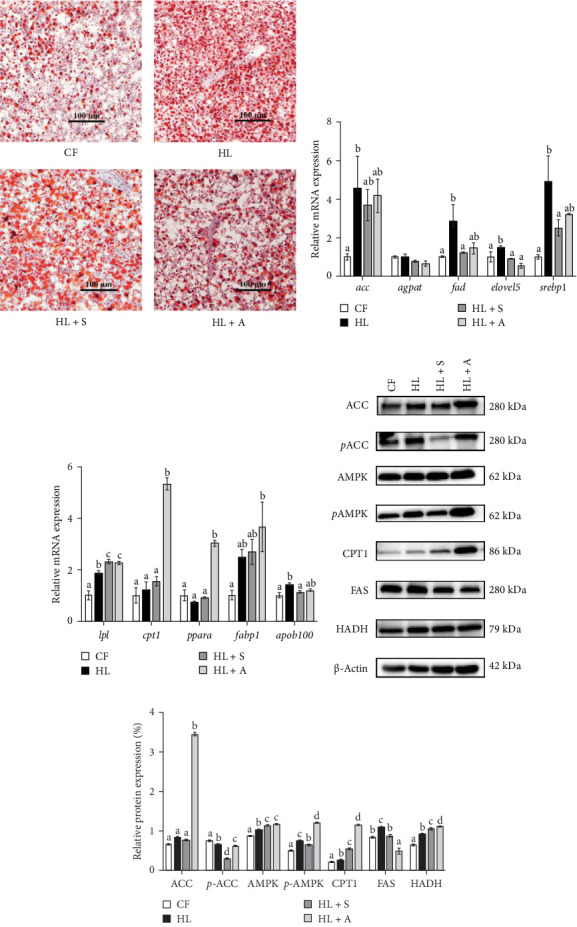
The effect of supplemental astaxanthin (AST) on hepatic lipid deposition and metabolism of *T. ovatus* fed with high lipid diet. (A) Hepatic lipid contents quantified by oil red O staining (100x, bars = 100 μm). (B) The mRNA expression of lipid anabolism relative genes of *T. ovatus*: *acc*, acetyl CoA carboxylase; *agpat3*, acylglycerol-3-phosphate acyltransferase; *fad*, fatty acid desaturases; *elovel5*, elongation of very long chain fatty acids protein 5; *srebp1*, sterol-regulatory element binding proteins 1. (C) The mRNA expression of lipid catabolism relative genes of *T. ovatus*: *lpl*, lipoprteinlipase; *cpt1*, carnitine palmotoyl transferase 1; *pparα*, peroxisome proliferators-activated receptors α; *fabp1*, fatty acid-binding protein 1; *apob100*, apolipoprotein B-100. (D) The representative bands of lipid metabolism of *T. ovatus*: *p*-ACC, phospho-Acetyl CoA carboxylase; AMPK, adenosine 5′-monophosphate-activated protein kinase; FAS, fatty Acid Synthase; HADA, hydroxyacyl-coenzyme A Dehydrogenase. (E) Densitometry analysis of immunoblots of Lipid metabolism rate-limiting enzymes expression in hepatic cell (*n* = 3). Results were presented as “mean ± SEM” of four replicates. Means with different superscripts letters are significantly different (*p* < 0.05).

To further substantiate these findings, quantitative real-time PCR was employed to evaluate the impact of different AST sources on lipid metabolism in *T. ovatus* fed a HL diet. The gene expression profiles in Figure 2B,C revealed a stark contrast in lipid metabolism-related genes between the experimental groups. Specifically, the HL group exhibited a significant upregulation of lipid anabolism genes compared to the CF group (*p* < 0.05), which was mitigated in the HL + S group. Remarkably, the HL + A group demonstrated the highest expression of lipid catabolism-related genes among all groups (*p* < 0.05). In detail, genes such as *acc*, fatty acid desaturases (*fad*), *elovl5*, and *srebp1* were significantly upregulated in the HL group versus the CF group (*p* < 0.05), with no significant difference between HL and HL + A groups (*p* > 0.05), but a significant downregulation in the HL + S group (*p* < 0.05). Conversely, the HL + A group displayed a markedly elevated expression of *cpt1*, peroxisome proliferators-activated receptors α complex (*pparα*), and *fabp1* compared to the other groups (*p* < 0.05), with no significant variations among CF, HL, and HL + S groups (*p* > 0.05).

To gain a more comprehensive understanding of AST's effects, western blotting was performed to assess the protein levels of key regulators involved in lipid metabolism. As illustrated in Figure 2C, both HL + S and HL + A groups effectively inhibited pACC compared to the CF and HL groups. Notably, the HL + A group exhibited significantly higher levels of CPT1 and pAMPK than all other groups (*p* < 0.05). Additionally, FAS expression was significantly downregulated in both HL + S and HL + A groups compared to the HL group (*p* < 0.05). These findings collectively underscore the potential of AST, regardless of its source, in positively modulating lipid metabolism and mitigating hepatic lipid accumulation in *T. ovatus*.

### 3.5. Hepatic Antioxidant Capacity Analysis

**Figure 3:**
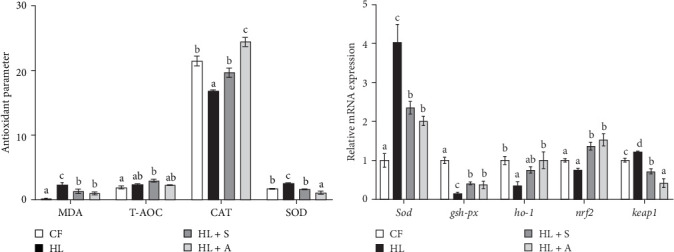
Hepatic antioxidant statuses of *T. ovatus* fed with or without high lipid and astaxanthin. (A) Antioxidant-related parameters in liver. MDA: malondialdehyde (nmol mg protein^−1^); T-AOC: T-AOC, total antioxidant capacity (mmol g protein^−1^) (B) Relative expression levels of antioxidant-related genes: *sod*, superoxide dismutase; *gsh-px*, glutathione peroxidase; *ho-1*, heme oxygenase 1; *nrf2*, nuclear factor erythroid-2-related factor 2; *keap1*, kelch-like ECH-associated protein 1. Results were presented as “mean ± SEM” of four replicates. Means with different superscripts letters are significantly different (*p* < 0.05).

The antioxidant profiles of the liver in *T. ovatus* are depicted in Figure 3. Specifically, Figure 3A reveals a notable elevation in both MDA and SOD levels within the HL group, significantly differing from the CF group (*p* < 0.05). However, this trend reverses in the HL + S and HL + A groups, where these levels decrease compared to the HL group (*p* < 0.05). In contrast, the CAT content in the liver of the HL diet-treated fish was notably lower than that of the CF diet-treated fish (*p* < 0.05), but it regained its levels in the HL + S and HL + A groups.

Subsequently, our attention shifted to the relative expression patterns of genes associated with antioxidant defense. The HL group exhibited a relatively high expression of *sod* and *keap1* mRNA, accompanied by a pronounced downregulation of *gsh-px*, *ho-1*, and *nrf2* compared to the CF group (*p* < 0.05). Notably, both the HL + S and HL + A groups displayed a similar trend, with their relative expression levels of antioxidant-related genes extremely higher than the HL group, suggesting that the inclusion of AST effectively enhanced the hepatic antioxidant capacity of *T. ovatus* subjected to a high-fat diet.

### 3.6. Inflammation, Apoptosis, and Immune Indexes Analysis

**Figure 4:**
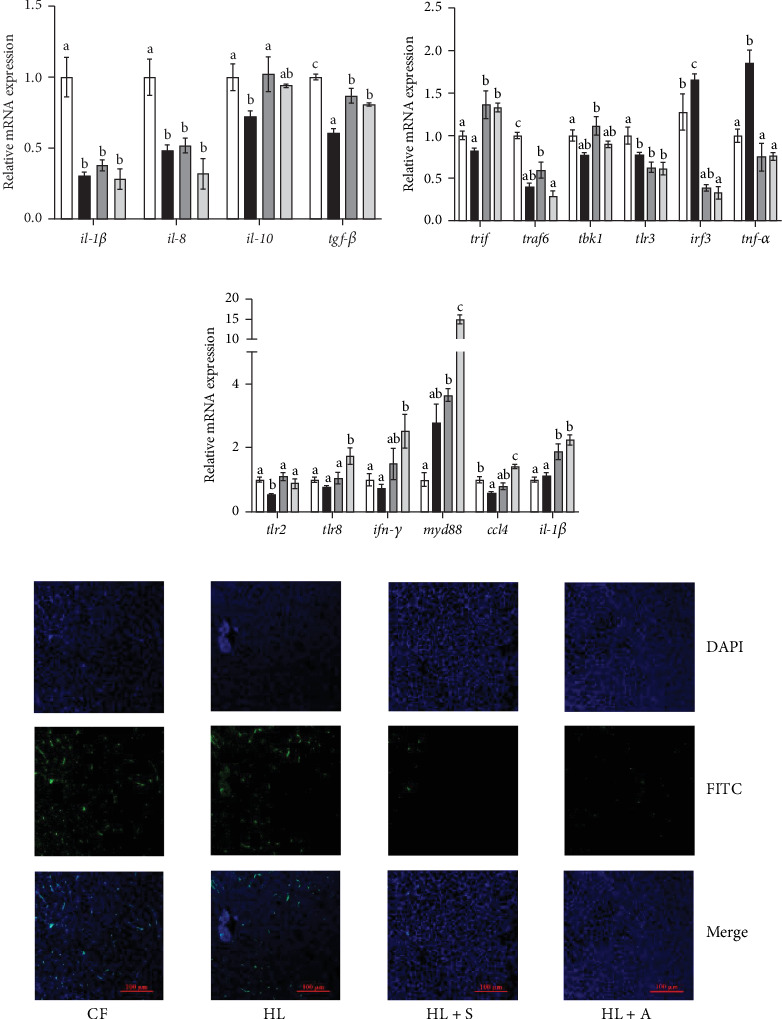
The effect of supplemental AST on inflammation, apoptosis, and immune indexes of *T. ovatus* fed with high lipid diet. (A) Relative expression levels of inflammation genes in liver: *il-1*β, interleukin-1β; *il-8*, interleukin-8; *il-10*, interleukin-10; *tgf-β*, transforming growth factor β. (B) Relative expression levels of inflammation-related genes in head kidney: *trif*, TIR domain-containing adaptor inducing interferon-β; *traf6*, tumor necrosis factor receptor-associated factors 6; *tbk1*, TANK-binding kinase 1; *tlr3*, toll-like receptor 3; *irf3*, interferon regulatory factor 3; *tnf-α*, tumor necrosis factor-α. (C) Relative expression levels of immune-related genes in head kidney: *tlr2*, toll-like receptor 2; *tlr8*, toll-like receptor 8; *ifn-γ*, interferon-γ; *myd88*, myeloid differentiation primary response protein 88; *ccl4*, chemokine (C–C motif) ligand 4. (D) TUNEL and DAPI combined staining in the liver of *T. ovatus*, first lines showed the corresponding fields of DAPI fluorescence, middle lines TUNEL fluorescence and final lines the merge fluorescence, as indicated. Original microscope magnification = 200x. Results were presented as “mean ± SEM” of four replicates. Means with different superscripts letters are significantly different (*p* < 0.05).

The inflammation, immune and apoptosis indexes in the liver and head kidney of *T. ovatus* was presented in Figure 4. As shown in Figure 4A, the expression levels of inflammation-related genes of liver in the HL group exhibited significant fluctuations compared to the CF group; but with the addition of AST, the gene expression levels in both the HL + S and HL + A groups tended to return to those observed in the CF group. In more detail, the expression of *il-1*β, *il-8*, *il-10*, and *tgf-β* declined enormously in HL group compared with other groups (*p* < 0.05).

To investigate the discrepancy in the effects of different sources of AST on the immune capacity of *T. ovatus* fed with a HL diet, we measured the expression of inflammation and immunity-related genes in the head kidney. According to Figure 4B and Figure 4C, the expression of anti-inflammatory genes (*trif*, *traf6*, *tbk1*) was significantly reduced in HL group compared to other groups, while pro-inflammatory genes (*irf3*, *tnf-α*) expression was markedly increased (*p* < 0.05). Furthermore, the expression levels of immune-related genes in the HL group are substantially lower than those in other groups, with the most pronounced difference observed in the HL + A group (*p* < 0.05).

The TUNEL and DAPI combined staining was experimentalized so as to investigate the diverse effect of different sources of AST alleviating apoptosis of hepatic cells in *T. ovatus* more intuitively. As the result denoted in Figure 4D, substantial apoptotic cells were captured in the images of CF and HL groups, while the number and size of apoptotic cells decreased strikingly in HL + S and HL + A groups compared with HL group. The reduction in apoptosis of hepatic cells suggests that both synthetic and algal AST protect the liver from fatal injury, thus providing more sturdy functions in growth, metabolism, immune, and antioxidant ability of *T. ovatus*.

## 4. Discussion

Lipids, serving as a dietary energy source, play a crucial role in fish growth. High utilization of dietary lipids is generally linked to protein sparing [23]. However, excessive lipid intake can have detrimental effects on fish health [12]. Based on our previous research findings, after an 8-week dietary intervention, the growth parameters such as WGR and SGR of *T. ovatus* fed a diet containing 150 g/kg of lipids without AST supplementation were markedly inferior to those observed in other groups [3]. This suggests that an excessive intake of dietary lipids, in the absence of AST, constrained the growth performance of *T. ovatus*. Similar reports regarding the inhibition of fish growth performance by HL diets have also been documented in other species such as *Oncorhynchus mykiss* [8], *Lateolabrax maculatus* [24], and *Megalobrama amblycephala* [25]. There are two primary reasons for the growth inhibition observed in fish when fed HL diets. First, the energy provided by a HL diet may exceed the growth requirements of the experimental fish, causing metabolic imbalances, reduced feed intake, and diminished utilization of other essential nutrients [26]. Second, excessive lipid intake may lead to fat accumulation in hepatic tissue, which results in metabolic disturbances and adversely affects liver health [27]. Consequently, it is crucial to develop a method to address these metabolic imbalances and mitigate oxidative damage to the liver induced by HL diets in *T. ovatus*. AST, as a potent antioxidant and potential immunomodulator, effectively functions as an additive to alleviate the negative effects associated with HL diets.

As elucidated by Han et al.[28], AST fosters enhanced nutrient utilization and growth in aquatic organisms through the modulation and regulation of their intermediary metabolic processes. Our prior investigation likewise demonstrated that the inclusion of either synthetic or algal AST in the diet significantly bolstered the growth performance of *T. ovatus* [29]. Consistent with these findings, recent research has reported similar benefits in other carnivorous fish species, such as *O. mykiss* [30] and *Sparus aurata* [31]. In the present study, we observed a marked suppression of growth in *T. ovatus* fed a HL diet, yet the introduction of AST significantly mitigated this inhibitory effect. Specifically, our data revealed a substantial reduction in WGR and SGR in the HL group, whereas the inclusion of either synthetic or algal AST restored these parameters to levels comparable to those of the CF group. This outcome not only reinforces previous conclusions but also conclusively establishes the capacity of AST to alleviate growth retardation induced by HL diets.

The hematological assessment of fish in aquaculture offers profound insights into their physiological well-being and overall health status [32]. When synergistically employed with other diagnostic routines, hematological analysis becomes a potent tool for identifying and evaluating stressors or diseases that may compromise production outcomes [33]. Notably, serum biomarkers such as triglycerides, cholesterol, and total protein are widely acknowledged indicators of animal health [34]. Moreover, alterations in serum TC, HDL-C, TG, and LDL-C levels, which serve as prognostic and diagnostic markers for liver health, are intimately tied to lipid metabolism in fish [35]. In the present study, *T. ovatus* fed a HL diet exhibited elevated serum LDL levels accompanied by notably lower serum glucose and HDL levels compared to the CF group. However, upon supplementing with either synthetic or algal-sourced AST, the content of TC, TG, and LDL decreased obviously from HL group levels, approaching those observed in the CF group. This trend aligns with findings from analogous studies on diverse fish species, including *Pseudosciaena crocea* [36], *Carassius auratus L*. [37] and *O. mykiss* [30], which have consistently reported that HL diets elevate serum TC, TG, and LDL while diminishing HDL levels, but these adverse effects are mitigated with AST supplementation. Collectively, these results underscore the effectiveness of dietary AST supplementation in alleviating lipid metabolism disruptions and hepatic hyperlipidemia in *T. ovatus* induced by HL diets.

In accordance with the aforementioned theory positing that a HL diet can potentially disrupt metabolic equilibrium, our primary research focus was directed towards exploring the alterations in lipid metabolism exhibited by fish administered with AST in conjunction with such a diet. To delineate the effects of AST, we initially employed macroscopic assessment methodologies. This involved calculating the VSI and HSI as quantitative measures of changes in body composition. Furthermore, we conducted a comprehensive analysis of fatty acid composition to uncover discrepancies potentially arising from different sources of astaxanthin. Additionally, we utilized liver oil red O staining to visually assess and quantify lipid accumulation within the liver tissue. In fish morphological indices, VSI and HSI indicated the physiological conditions through the method of mirroring the nutritional status [38]. In the present study, fish fed a HL diet exhibited elevated HSI and VSI values, whereas the inclusion of both synthetic and algal-sourced AST in the HL diet mitigated these elevations in *T. ovatus*. The fatty acid composition is a crucial indicator of the nutritional value of fish, which can significantly influence its market price [11]. In addition, LC-PUFA plays essential and complex physiological roles in fish, including maintaining cell membrane structure and fluidity, transporting plasma triglycerides, efficiently absorbing fat-soluble vitamins in the intestines, and regulating transcription, cell functions, and signaling pathways [39, 40]. Moreover, the variation of fatty acid profiles in muscle suggested that HL diet can affect the fatty acid profiled of the *T. ovatus*. High-fat diets, with or without AST, elevated the levels of unsaturated fatty acids, particularly the n-3 and n-6 series. Notably, the supplementation with algal-sourced AST resulted in the highest concentrations of these beneficial fatty acids. Similar results were also reported in some teleost such as *O. mykiss* [41], *C. auratus L*. [37], and *Salmo salar L*. [42]. Research indicates that *H. pluvialis* is abundant in polyunsaturated fatty acids (PUFAs), encompassing valuable components such as linoleic acid (C18:3), docosahexaenoic acid (DHA), and eicosapentaenoic acid (EPA) [43]. Based on the results of muscle fatty acid composition, it can be concluded that the changes in dietary fatty acid content caused by high-fat and AST diets can alter the fatty acid composition of the muscle of *T. ovatus*, thereby increasing the content of PUFAs to enhance the quality of the fish meat. Oil red O staining, recognized as the most direct indicator of liver lipid deposition [44], permits direct observation of hepatic lipid metabolism alterations due to HL diets and exploration of AST's potential to mitigate abnormal lipid accumulation in fish consuming such diets. The sharp increase in the number and size of lipid droplets in the HL group, coupled with the decrease observed in the HL + S and HL + A groups, collectively validates the possibility that AST may reduce excessive lipid accumulation in the liver by regulating lipid metabolism, thereby alleviating liver burden and decreasing the risk of fatty liver disease. Consistent with these results, previous studies have reported that long-term feeding with a HL diet can significantly promote lipid accumulation in fish liver, such as *Ctenopharyngodon idella* [44], *Cyprinus carpio L*. [45] and *Larmichthys crocea* [46], resulting in higher levels of HSI, VSI, and an increase in the number and size of lipid droplets in the liver. Nevertheless, these phenomena can be well improved by adding AST to the feed, as evidenced in studies on *M. salmoides* [47], *O. mykiss* [41], and *M. amblycephala* [25]. These results clearly demonstrate that both synthetic and algal AST can effectively reduce excessive lipid accumulation, thereby lessening metabolic stress in the liver and likely reducing oxidative stress in the liver.

Subsequently, we detected the expression of genes and proteins related to lipid anabolism and catabolism, including using qPCR to detect the expression of related genes and the Western Blot method to show protein expression levels, in order to further analyze the mechanism by which AST reduces excessive lipid accumulation caused by a HL diet. The process of De novo lipogenesis (DNL) within the liver emerges as a pivotal factor influencing lipid deposition, with ACC occupying a central stage in the regulation of fatty acid synthesis and oxidation, ultimately dictating the extent of hepatic lipid accumulation [48]. FAS and ACC are key rate-limiting enzymes in fatty acid synthesis, and sterol-regulatory element binding proteins 1 (SREBP-1), a nuclear transcription factor, is crucial in regulating fatty acid synthesis and acts as a key regulator of the rate-limiting enzymes FAS and ACC [49]. Additionally, p-AMPK inhibits SREBP-1 cleavage and nuclear translocation, which in turn suppresses SREBP-1 mediated target gene expression in hepatocytes after fat overaccumulation, leading to reduced lipid accumulation [50]. Besides, the capacity to decompose fatty acids also influences the extent of lipid deposition in the liver. Increased lipid levels in hepatocytes activate fatty acid β-oxidation through upregulation ofPPARα, and adenosine 5′-AMPK upregulates lipolysis marker, lipoprotein lipase (LPL) and CPT1 to maintain lipid homeostasis during energy stress [51]. In this study, the hepatic expressions of *acc*, *fad*, elongation of very long chain fatty acids protein 5 (*elovel5*), *srebp1*, *lpl*, fatty acid-binding protein 1 (*fabp1*) and apolipoprotein B-100 (*apob100*) in HL group was significantly higher than those in CF, indicated that fish fed with HL diet indeed suffered from the stress of excessive lipid deposition in liver and were thirsty for reducing the content of lipid by upregulating lipid catabolism. However, with the addition of AST, this disorder has been effectively alleviated. In comparison to the HL group, the HL + S group showed a greater reduction in the expression of *acc*, *fad*, and *srebp1*, while the HL + A group demonstrated higher expression levels of *cpt1*, *pparα*, and *fabp1*. The WB results further validated this trend, revealing a decrease on *p*ACC levels in HL + S group and on FAS level in HL + A group, while the levels of CPT1, AMPK and *p*AMPK in the HL + A and HL + S groups were significantly higher compared to the CF group. Interestingly, in this study, the HL group showed a trend akin to the two AST addition groups in promoting lipid catabolism and inhibiting lipid anabolism, which can be considered as a self-regulation mechanism of the fish body. Nonetheless, this inherent protective mechanism alone is inadequate to fully counteract the lipid metabolism disruptions caused by excessive lipid intake. It is only with the inclusion of AST that these negative impacts are effectively alleviated. Our previous study has shown that activating the AMPK/ACC/CPT1 signaling pathway while inhibiting the AMPK/SREBP-1/FAS pathway is of paramount importance, potentially enabling the alleviation of lipid deposition in hepatic tissues resulting from a HL diet [8]. The present investigation elucidates that AST demonstrates a potent capability to mitigate excessive lipid accumulation in the liver by facilitating lipid catabolism and suppressing lipid anabolic processes.

The consumption of a HL diet is recognized to elicit lipid deposition, which subsequently initiates oxidative stress, enhances lipid peroxidation, and elevates ROS production, ultimately compromising organelle stability and disrupting cellular operations [8]. Serving as one of the antioxidant defense mechanisms in fish, the enzymatic antioxidant defense mechanism comprises a diverse array of enzymes, including CAT and SOD, with the major function of mitigating excessive ROS levels [18, 52]. Furthermore, the antioxidant profile and efficacy in fish can be accurately gauged using the vital biomarkers MDA and T-AOC. MDA, which arises as a consequence of lipid peroxidation, acts as a proxy for the intensity of this peroxidation process, with elevated levels posing risks to protein functionality and cellular structural integrity [53]. The Nrf2-ARE pathway is also crucial in protecting cells from oxidative stress by neutralizing reactive oxidants [54]. Upon encountering stress signals, the cytoplasmic Nrf2-Keap1 complex undergoes separation, allowing Nrf2 to journey to the nucleus. Within the nucleus, Nrf2 attaches to the ARE, setting off a cascade of events that leads to the transcriptional induction of several downstream antioxidant enzyme genes, such as *gr*, *ho-1*, and *mn-sod* [55]. Based on the assessment of hepatic antioxidant capacity, our study revealed that the HL diet significantly induced oxidative stress in the liver of *T. ovatus* when AST was absent, accompanied by suppression of the primary antioxidant defenses. However, the inclusion of AST in the HL diet reversed these abnormal perturbations. To further elucidate the underlying molecular mechanisms, we evaluated the expression levels of genes involved in the Nrf-Keap1 antioxidant signaling pathway. The results indicated that the Nrf2-Keap1 signaling pathway was attenuated in the HL group, leading to downregulation of its downstream effectors, *gsh-px* and *ho-1*. Consequently, there was an increased reliance on *sod* expression to counteract the oxidative stress induced by the high-fat diet. Notably, the incorporation of AST, particularly algal-derived AST, into the diet restored the suppressed Nrf2-Keap1 pathway, resulting in an upregulation of *gsh-px* and *ho-1* levels. As a result, the liver reduced its dependence on *sod* expression and mitigated hepatic stress. Consistent with our findings, similar results have been reported in *P. crocea* [25] and *O. mykiss* [56], demonstrating that both synthetic and algal AST supplementation in the diet can enhance the antioxidant capacity of carnivorous fish. The present study further confirms that dietary supplementation with two sources of AST improves the antioxidant capacity of *T. ovatus* by activating the Nrf2-ARE signaling pathway.

The nonspecific immune and antioxidant systems constitute the primary defenses against pathogen invasion in fish [57]. Furthermore, hepatic lipotoxicity arising from deregulated lipid metabolism triggers hepatocellular inflammation and programed cell death [58]. To assess the protective effects of two forms of AST on *T. ovatus* fed a HL diet, we investigated the indices of apoptosis, immunity, and inflammation. Specifically, we measured the expression levels of pro-inflammatory factor genes in the liver and inflammatory and immune-regulatory genes in the head kidney. Consistent with our findings on antioxidant properties, supplementing with synthetic AST significantly upregulated the expression of genes promoting liver inflammation (such as *il-1β*, *il-8*, and *tgf-β*) compared to fish fed the HL diet alone. Various studies have demonstrated that viral proteins target TLR3 or TRIF to suppress the host's interferon (IFN) response via distinct mechanisms [59]. In fish, TLR3 plays a pivotal role in the innate immune system by recognizing double-stranded RNA (dsRNA) and activating the downstream TRIF pathway. This activation involves TRIF binding to the toll/interleukin-1 receptor (TIR) domain of TLR3, ultimately triggering IFN promoter activation in response to poly I: C [60]. Similarly, inhibitors interacting with TANK-binding kinase 1 (TBK1) in TLR3-mediated signaling can markedly suppress downstream molecules, including interferon regulatory factor 3 (IRF3), IFN-β, and tumor necrosis factor-α (TNF-α). These observations suggest that TRIF, TNF receptor-associated factor 6 (TRAF6), and TBK1 are indispensable for the activation of TLR3-mediated signaling [61]. In our study, synthetic AST significantly upregulated the expression of anti-inflammatory genes (such as *trif*, *traf6*, and *tbk1*) and downregulated the expression of inflammatory cytokines in the head kidney. Conversely, algal AST markedly increased the levels of genes associated with innate immunity (such as *tlr2*, *ifn-γ*, and *ccl4*) compared to fish fed the HL diet without AST. Furthermore, TUNEL staining results corroborated our previous findings, demonstrating that both sources of AST can effectively ameliorate the oxidative stress and apoptosis induced by the HL diet in the liver. In conclusion, synthetic AST mitigates the detrimental effects of a HL diet on fish by modulating inflammation both locally in the liver and systemically. Meanwhile, algal AST enhances immune capacity and reduces cellular apoptosis, effectively preempting the potential harms posed by a HL diet at its source in *T. ovatus*.

## 5. Conclusion

In summary, the growth performance of fish exhibited significant decline, accompanied by an increase in VSI and HSI, when subjected to HL diets. However, fish fed with diets supplemented with HL + S and HL + A reversed these adverse effects. These dietary interventions not only enhanced growth performance but also alleviated the elevated physiological indices. Furthermore, both sources of AST mitigated hepatic lipid deposition by modulating the size and number of lipid droplets, adjusting fatty acid composition, and improving lipid metabolism. Consequently, the reduction in lipid overaccumulation led to decreased oxidative stress in the liver, an effect that was further potentiated by the enhanced hepatic antioxidant defenses provided by AST supplementation. Last, this study confirmed the immune-enhancing and anti-inflammatory properties of AST, which corroborated the aforementioned findings and collectively demonstrated that the inclusion of AST effectively mitigates the detrimental consequences associated with HL diets. The findings derived from this study offer substantial theoretical underpinning and robust data support for the advancement of high-fat feed formulations tailored for fish nutrition.

## Figures and Tables

**Table 1:** Ingredients and proximate composition of the experimental diets (% dry matter).

Ingredients	CF	HL	HL + S	HL + A
Soybean meal	30.84	21.45	21.45	21.45
Wheat flour	22.73	20.85	20.83	20.81
Red Fish meal	18	15	15	15
Chicken meal	10.5	15	15	15
Soy protein concentrate	0	5	5	5
Cassava starch	1	2	2	2
Fish oil	3.3	4	4	4
Soybean oil	6	8.93	8.93	8.93
Soya lecithin	2	2	2	2
Synthetic Astaxanthin	0	0	0.02	0
Algal Astaxanthin	0	0	0	0.04
Ca(H2PO4)2	1	1	1	1
Multivitamin	1	1	1	1
Multimineral	1	1	1	1
Choline chloride	0.5	0.5	0.5	0.5
Vitamin C	0.5	0.5	0.5	0.5
DL-Met	0.43	0.45	0.45	0.45
Lys-HCL (99%)	0.78	0.79	0.79	0.79
Threonine	0.42	0.53	0.53	0.53
Sum	100	100	100	100
Nutrient levels	—	—	—	—
Moisture	9.7	8	8	8
Crude protein	39.31	39.66	39.3	39.24
Crude lipid	10.92	16.36	16.55	17.05

**Table 2:** Sequences of primers used in this study.

Gene name	Primer sequence (5′-3′)	Accession
*acc*	F-GTTGTCAATCCCAGCCGATC	Zhou et al. [15]
	R-ATCCACAATGTAGGCCCCAA	
*agpat*	F-CTTCCTGTTTTGGGCCACTC	Li et al. [16]
	R-GTCGCCATAACTTGAGCCTG	
*fad*	F-GAACAATCCCACTTCAACG	Zhu et al. [17]
	R-AGGAATCCCATACTTCTCACA	
*elovel5*	F-TACATGGTCACGCTCATTATCC	Zhu et al. [17]
	R-CCGTTCTGATGCTCCTTCTTTA	
*srebp1*	F-GAGCCAAGACAGAGGAGTGT	Zhou et al. [15]
	R-GTCCTCTTGTCTCCCAGCTT	
*lpl*	F-TTTGTCCTTCCTCGTCACCA	Li et al. [16]
	R-AAGACAGCATCCTCTCCACC	
*cpt1*	F-CAAATCCACCACCAGCAC	Fang et al. [3]
	R-GGAAGGCAGCAGATAAAC	
*pparα*	F-AATCTCAGCGTGTCGTCTT	Fang et al. [3]
	R-GGAAATGCTTCGGATACTTG	
*fabp1*	F-CCAAGGACATCAAGCCAATTAC	Li et al. [16]
	R-TGGTGATTTCAGCCTCCTTAC	
*apob100*	F-AAAAGCCACAAGACGAAAGCA	Li et al. [18]
	R-GAAGCAGCAAAAGGCAGAGC	
*sod*	F-CCTCATCCCCCTGCTTGGTA	Fang et al. [3]
	R-CCAGGGAGGGATGAGAGGTG	
*gsh-px*	F-GCTGAGAGGCTGGTGCAAGTG	Fang et al. [3]
	R-TTCAAGCGTTACAGCAGGAGGTTC	
*ho-1*	F-AGAAGATTCAGACAGCAGCAGAACAG	Fang et al. [3]
	R-TCATACAGCGAGCACAGGAGGAG	
*nrf2*	F-TTGCCTGGACACAACTGCTGTTAC	Fang et al. [3]
	R-TCTGTGACGGTGGCAGTGGAC	
*keap1*	F-CAGATAGACAGCGTGGTGAAGGC	Fang et al. [3]
	R-GACAGTGAGACAGGTTGAAGAACTCC	
*il-1*β	F-CGGACTCGAACGTGGTCACATTC	Zhao et al. [8]
	R-AATATGGAAGGCAACCGTGCTCAG	
*il-8*	F-TGCATCACCACGGTGAAAAA	Zhao et al. [8]
	R-GCATCAGGGTCCAGACAAATC	
*il-10*	F-CTCCAGACAGAAGACTCCAGCA	Zhao et al. [8]
	R-GGAATCCCTCCACAAAACGAC	
*tgf-β*	F-GAGATACGGAAAAGAGTGGGG	Zhao et al. [8]
	R-TGACAAAGCGGGAAGCAAG	
*trif*	F-GGTGGCAAATGAGTGGAGGTAA	He et al. [19]
	R-GCTGTCAGTGGAAAGGGCTCT	
*traf6*	F-TGATGGCTTTGAGGAATGCTG	He et al. [19]
	R-ACAGAATCTCCCGTTTGGCA	
*tbk1*	F-GCATCGTTCATCGGGACATC	He et al. [19]
	R-CAGAGACACAAACTGCTCGTCG	
*tlr3*	F-CAGCCACCTCAGTCTCAA	Wu et al. [20]
	R-ATCACCACCAGTCTGTTGT	
*irf3*	F-ACAAGAACGAAACCGCTAACCC	Wu et al. [20]
	R-TCATCAAAGCACGAGACCACC	
*tnf-α*	F-GCTCCTCACCCACACCATCA	Zhao et al. [8]
	R-CCAAAGTAGACCTGCCCAGACT	
*tlr2*	F-CTCCACCTTGCGATACCT	Wu et al. [19]
	R-TCCAACACCTCCAGAGATG	
*tlr8*	F-TGGGTGATGAGAAATCTGCG	Wu et al. [20]
	R-GCCTCTGTTAAGACAAAAAGGG	
*ifn-γ*	F-GTTGGAAGTGGGCGAGGAT	Zhu et al. [21]
	R-TTTGTTCTGGACGACGAGGTT	
*myd88*	F-AATACCTTGACAGCGATGCCTG	Fang et al. [3]
	R-GTGCAAGGCCTGGTGTAATCA	
*ccl4*	F-TTTGTGCTGATGCTGGCTTTC	Sun et al. [22]
	R-CCGCTGGCTGGTCTTGATG	

**Table 3:** Growth performance and feed utilization of *T. ovatus* fed with or without high lipid and astaxanthin diets.

	CF	HL	HL + S	HL + A
IBW	4.21 ± 0.03	4.17 ± 0.06	4.15 ± 0.08	4.18 ± 0.06
WGR	345.46 ± 2.55^b^	315.37 ± 4.85^a^	334.99 ± 7.32^b^	342.35 ± 14.69^b^
SGR	2.87 ± 0.01^b^	2.55 ± 0.08^a^	2.89 ± 0.08^b^	2.82 ± 0.09^b^
SR	85.00 ± 1.44^a^	85.00 ± 1.44^a^	94.13 ± 0.59^b^	89.13 ± 1.18^b^
VSI	6.65 ± 0.25^a^	8.84 ± 0.34^c^	7.55 ± 0.25^b^	6.89 ± 0.26^ab^
HSI	1.51 ± 0.10^a^	2.23 ± 0.18^b^	1.78 ± 0.18^a^	1.38 ± 0.11^a^

*Note:* Initial body weight (FBW, g) = initial body weight/initial number of fish; weight gain ratio (WGR, %) = 100 × (final individual weight − initial individual weight)/initial individual weight; specific growth rate (SGR, %/day) = 100 × (Ln (final individual weight) − Ln (initial individual weight)) / number of feeding days; survival rate (SR, %) = 100 × (final number of fish)/(initial number of fish); viscerosomatic index (VSI, %) = 100 × (viscera weight, g) / (whole bodyweight, g); hepatosomatic index (HSI, %) = 100 × (liver weight, g)/(whole body weight, g). Results were presented as “mean ± SEM” of four replicates, and mean values on the same line with different superscript letters denoted significantly different (*p* < 0.05), while with the same letter or no letter indicated no significant difference (*p* > 0.05).

**Table 4:** Fatty acid composition (% total fatty acids) in the muscle of *T. ovatus* fed with experimental diets.

Ingredient	CF	HL	HL + S	HL + A
Fatty acid composition (% total fatty acids)				
C12:0	0.03 ± 0.00^a^	0.03 ± 0.00^b^	0.00 ± 0.00^c^	0.00 ± 0.00^c^
C14:0	1.50 ± 0.02^a^	1.46 ± 0.00^b^	1.45 ± 0.00^b^	1.42 ± 0.01^c^
C16:0	20.11 ± 0.02^a^	18.32 ± 0.03^c^	19.84 ± 0.02^b^	17.32 ± 0.06^d^
C16:1	2.55 ± 0.00^a^	2.55 ± 0.00^b^	2.55 ± 0.00^c^	2.55 ± 0.00^d^
C18:0	6.12 ± 0.00^a^	5.54 ± 0.00^d^	5.96 ± 0.00^b^	5.65 ± 0.00^c^
C18:1	25.38 ± 0.00^a^	24.60 ± 0.01^c^	25.09 ± 0.02^b^	24.08 ± 0.00^d^
C18:2	29.65 ± 0.04^a^	32.71 ± 0.03^c^	30.28 ± 0.02^b^	33.43 ± 0.03^d^
C20:0	0.52 ± 0.00^b^	0.52 ± 0.00^a^	0.49 ± 0.00^d^	0.51 ± 0.00^c^
C18:3*n*3	2.47 ± 0.00^a^	2.77 ± 0.01^b^	2.48 ± 0.01^a^	2.86 ± 0.00^c^
C20:1	1.04 ± 0.00^a^	0.94 ± 0.00^c^	0.99 ± 0.00^b^	0.89 ± 0.00^d^
C20:2	1.80 ± 0.00^b^	1.79 ± 0.00^c^	1.79 ± 0.00^d^	1.86 ± 0.00^a^
C20:3*n*6	0.21 ± 0.00^a^	0.21 ± 0.00^b^	0.19 ± 0.00^c^	0.18 ± 0.00^d^
C22:0	0.37 ± 0.00^b^	0.35 ± 0.00^a^	0.37 ± 0.00^b^	0.39 ± 0.00^c^
C20:3*n*3	0.38 ± 0.00^a^	0.42 ± 0.00^c^	0.39 ± 0.00^b^	0.47 ± 0.00^d^
C20:4	0.61 ± 0.00^a^	0.62 ± 0.00^b^	0.62 ± 0.00^b^	0.66 ± 0.00^c^
C22:1	0.27 ± 0.00^b^	0.24 ± 0.00^a^	0.28 ± 0.00^c^	0.25 ± 0.00^a^
C22:2	0.32 ± 0.00^c^	0.30 ± 0.00^a^	0.32 ± 0.00^b^	0.33 ± 0.00^c^
C20:5	0.69 ± 0.00^a^	0.68 ± 0.00^a^	0.74 ± 0.00^b^	0.76 ± 0.00^c^
C22:6	4.25 ± 0.01^a^	4.20 ± 0.01^a^	4.57 ± 0.02^b^	4.64 ± 0.02^c^
C22:5*n*6	0.32 ± 0.00^c^	0.30 ± 0.00^a^	0.32 ± 0.00^b^	0.32 ± 0.00^c^
C22:5*n*3	0.82 ± 0.00^b^	0.90 ± 0.00^c^	0.81 ± 0.00^a^	0.97 ± 0.00^d^
Others	0.58 ± 0.00^c^	0.57 ± 0.00^a^	0.58 ± 0.00^b^	0.59 ± 0.00^d^
∑SFAs	29.23 ± 0.03^a^	26.79 ± 0.05^c^	28.69 ± 0.02^b^	25.89 ± 0.06^d^
∑MUFAs	29.25 ± 0.00^a^	28.31 ± 0.01^c^	28.81 ± 0.02^b^	27.64 ± 0.00^d^
∑PUFAs	41.52 ± 0.04^a^	44.90 ± 0.05^c^	42.50 ± 0.04^b^	46.48 ± 0.06^d^
*n−*3	8.61 ± 0.01^a^	8.97 ± 0.02^b^	8.99 ± 0.02^b^	9.69 ± 0.03^c^
*n−*6	32.91 ± 0.03^a^	35.93 ± 0.03^c^	33.51 ± 0.02^b^	36.78 ± 0.03^d^
*n−*3/*n−*6	0.26 ± 0.00^b^	0.25 ± 0.00^a^	0.27 ± 0.00^d^	0.26 ± 0.00^c^

*Note:* Results were presented as “mean ± SEM” of four replicates, and mean values on the same line with different letters denoted significantly different (*p* < 0.05), while with the same letter or no letter indicated no significant difference (*p* > 0.05).

## Data Availability

The data are available upon request from the authors.

## Supporting Information

Supporting Information Our manuscript reported on animal experiments, so we followed the ARRIVE reporting guidelines and provided a summary of our animal experiments called Author Checklist so that editors, reviewers, and readers could assess the reliability of the finding.
